# A Descriptive Cross-Sectional Study of Manipulative Dexterity on Different Subtypes of Multiple Sclerosis

**DOI:** 10.1155/2020/6193938

**Published:** 2020-04-28

**Authors:** Elisabet Huertas-Hoyas, Nuria Máximo-Bocanegra, Carlos Diaz-Toro, Raquel Montes-Diez, Jorge Pérez-Corrales, Patricia Sánchez-Herrera-Baeza, Rosa Mª Martínez-Piédrola, Cristina García-Bravo, Carlos Sánchez-Camarero, Marta Pérez-de-Heredia-Torres

**Affiliations:** ^1^Rehabilitation and Physical Medicine Department, Rey Juan Carlos University, Avenida de Atenas s/n. CP.28922, Alcorcón, Madrid, Spain; ^2^Residencia Municipal y Centro de Día “Villa Elena”, Spain; ^3^Computing Science, Computer Architecture, Programming Languages and Systems, and Statistics and Operational Research Department, Rey Juan Carlos University, Tulipán s/n. CP.28933, Móstoles, Madrid, Spain

## Abstract

**Background:**

Manipulative dexterity impairments affect 76% of individuals with multiple sclerosis (MS). Patients with MS can experience reduced skill when performing both basic activities of daily living and instrumental activities of daily living. Many studies consider that physical rehabilitation leads to a decrease in the level of disability, especially at the mild and moderate stages of the disease. However, most studies fail to distinguish between the different MS subtypes.

**Purpose:**

Our aims were (1) to describe the manipulative skills of people according to the different subtypes of MS, (2) to analyze the correlation between dexterity and self-perception variables, and (3) to identify possible predictors of functionality. *Study Design*. A descriptive cross-sectional study.

**Methods:**

30 individuals with MS. The measurement tools used were the ABILHAND, the Purdue Pegboard Test (PPT), the Nine-Hole Peg Test, and the Box and Block Test.

**Results:**

All subtypes of MS obtained lower scores for manipulative dexterity compared to normal skill levels, with individuals with primary progressive MS displaying the lowest values. However, the analysis of differences between the different subtypes did not reveal statistically significant intragroup differences. In addition, differences were found regarding the effect size of practically all the variables analyzed for both manipulative dexterity (PPT, NHPT, and BBT) and the self-perception of ADLs (ABILHAND), for which high values (*d*_*r*_ = 0.72) and very high values (*d*_*r*_ = 1.46) were obtained.

**Conclusions:**

Although no significant differences were found between the different types of MS, the assessment of manual dexterity and perceived efficacy of daily activities must be considered as prognostic factors in the progression of the disease. These findings may help support further research on targeted interventions to improve dexterity deficiencies, as well as promote an improved quality of therapeutic interventions.

## 1. Introduction

Multiple sclerosis (MS) is a chronic neurological disease. According to the evolution of the disease, MS is classified into different subtypes: relapsing-remitting MS (RRMS), secondary progressive MS (SPMS), and primary progressive MS (PPMS) [1]. An initial classification included a progressive relapsing subtype (PR); however, this term was later abandoned because it overlapped with other subtypes [2]. These terms are only clinical descriptors, and it remains uncertain whether they are able to distinguish among potentially different disease mechanisms. The natural clinical history and prognostic features of typical RRMS have been well defined by population-based studies with long follow-up periods. In the case of SPMS, a secondary progressive phase follows an exacerbating and remitting course based on a consensus definition [3]. Thus, SPMS is diagnosed retrospectively by a history of gradual worsening after an initial relapsing disease course, with or without acute exacerbations during the progressive course of the disease. To date, there are no clear clinical, imaging, immunologic, or pathologic criteria to determine the transition point when RRMS converts to SPMS, as this transition is usually gradual [2]. The term PPMS is now used to describe the progressive phase of the disease. Patients with PPMS have always represented a substantial minority in cross-sectional or longitudinal studies of multiple sclerosis populations. This includes research conducted at the clinic or hospital or autopsy-based. Only recently, cases of PPMS have received greater attention. This is thanks to advances in diagnostic imaging and cerebrospinal fluid studies which have largely eliminated the diagnostic difficulty that has accompanied this somewhat atypical presentation. Nevertheless, there is still an extended delay between the first symptom and diagnostic confirmation of the disease [4].

A large body of evidence supports physical rehabilitation and the pharmacological treatment of people with RRMS; however, this is more limited in the specific case of PPMS or SPMS, due to their distinct characteristics. Thus, as stressed by the International Progressive Multiple Sclerosis Alliance, there is a need to focus on increasing research and funding for PPMS [5].

The common clinical signs of the disease include motor and sensory deficits, cerebellar symptoms, fatigue, and/or vision problems [6], which can impact manipulative dexterity, among other significant aspects. Specifically, manipulative dexterity is impaired in 76% of the population with MS [7]. Often, patients also present bilateral impairments [8, 9].

The motor deficits of MS hamper the performance of activities of daily living (ADLs), resulting in a decline of independence and quality of life [10]. The occupational performance (OP) of patients with MS is up to 50% less when compared to people without neurological pathology. This is also reflected in subjective questionnaires on the perceived use of the UL [9]. Patients with MS, at both the moderate and severe stages of the disease, can experience reduced skill when performing both basic activities of daily living (BADL) and instrumental activities of daily living (IADL). Although, in some cases, patients may be independent in BADL, the performance of IADL can still be affected and patients may not be able to perform these satisfactorily [11].

Among the assessment scales available for the measurement of manipulative dexterity, the Nine-Hole Peg Test (NHPT) is highlighted as being a good predictor of functionality [12, 13]. In addition, this test may serve to identify people with activity limitations and participation restrictions [14]. Regarding dominance, the nondominant UL usually obtains significantly worse scores in all tests for the assessment of manipulative dexterity when compared to the dominant UL. A great majority of UL rehabilitation strategies in people with MS are based on the dominant UL or on the more affected UL, although most ADLs are performed bilaterally in a coordinated manner [5]. Another variable which has an impact on manipulative dexterity is the use of a wheelchair, as this is negatively correlated with the use of the UL in ADLs [15].

Many studies consider their results in terms of the level of disability, highlighting that physical rehabilitation leads to improvements, especially at the mild and moderate stages of disability, rather than at the more severe stages [16, 17]. However, most studies do not distinguish between the different MS subtypes [18]. There is also an inherent tendency to overlap PPMS and RRMS or SPMS even though the clinical manifestations of the former generally develop far earlier and with a greater intensity, leading to higher levels of disability [19]. Despite these results, the functional reorganization of the brain and neuroplasticity processes are well described in all subtypes of MS. Thus, an increased activation and recruitment of additional areas of the central nervous system and an improvement of certain motor networks have been shown to occur with training and rehabilitation, meaning that patients benefit from a multidisciplinary approach [20]. To gain deeper knowledge of manipulative dexterity impairments in MS and to provide greater clarity on which therapies are most appropriate for the different disease subtypes, this study sought to describe the manipulative dexterity of the different types of multiple sclerosis (PPMS, RRMS, and SPMS) and to analyze the correlations among dexterity variables and self-perception and to identify the possible predictors of functionality.

## 2. Material and Methods

### 2.1. Study Design

A cross-sectional descriptive study design was employed. After voluntarily signing informed consent, 35 patients with MS were recruited. The group was analyzed first by the type of MS: (a) RRMS, (b) PPMS, and (c) SPMS and secondly according to the most affected side of the body: dominant or nondominant. The assessment was performed by five professionals, all of whom were occupational therapists.

### 2.2. Sample

This study was conducted in the Autonomous Community of Madrid, Spain. The study sample was obtained by using nonprobabilistic convenience sampling over a three-month patient recruitment period. Consecutive sampling was performed on a group of 35 volunteer participants from different MS care centers: The Multiple Sclerosis Association of Valdemoro, The Multiple Sclerosis Association of Móstoles, and the FOREN Rehabilitation Center. The following selection criteria were established: people diagnosed with MS, with no flare-ups during, at least, the three previous months; not presenting cognitive decline (measured using the Montreal Cognitive Assessment (MoCA)); aged between 25 and 60 years; with a score of six or lower on the Expanded Disability Status Scale (EDSS), and regularly attending physiotherapy and/or occupational therapy rehabilitation treatment.

### 2.3. Assessment Tools

The following assessment scales were used to measure manipulative dexterity:

- *Expanded Disability Status Scale (EDSS)* [21]. Designed by John Kurztke in 1955 and revised and expanded in 1983, this tool is the most widely used MS scale. The EDSS quantifies disability based on eight functional systems and enables professionals to assign a level for each functional system. Despite the widespread use of this scale, there are several limitations and difficulties related to the use of the same, meaning that it is important to exercise caution when interpreting the results of clinical protocols that use this scale as a measurement tool
- *Montreal Cognitive Assessment (MoCA)*. Developed by Ziad Nasreddine in 1996 [22], this assessment explores cognitive functions of attention, concentration, executive functions, memory, language, conceptual thought, calculation, and orientation. The administration time is approximately 10.15 minutes, depending on the patient's capacity, with a maximum score of 26
- *Nine-Hole Peg Test (NHPT)* [12]. This test assesses eye-hand coordination and fine motor motricity, among other aspects. It contains nine pegs which must be placed and removed as fast as possible into nine holes on a board. This test is performed with each UL, and the execution time for each is measured
- *Box and Block Test (BBT)* [23]. This test evaluates motor components, such as gross motor mobility, eye hand coordination, or crossing the midline. This test measures the number of cubes that the patient is able to move from one compartment to the other with each UL in one minute
- *Purdue Pegboard Test (PPT)* [24]. This test assesses aspects that are related to manipulative dexterity, such as gross motor mobility or bilateral coordination. There are four different tests: three 30-second tests, one for each UL, and another bimanual test, together with a last one-minute test of bimanual assembly (counting the washers, pins, and collars inserted)
- *ABILHAND* [25]. This test is based on an interview that scores the perception that a person has regarding the performance of different ADLs, with four possible scores: impossible, hard, easy, and never performed. The approximate administration time ranges between 5 and 10 minutes

### 2.4. Procedure

The assessment tools were administered in the same consecutive order as the participants were recruited. According to the current legislation regarding ethical aspects in research, information regarding this study was provided to each participant, together with the informed consent. The study was designed according to the ethical norms of the Helsinki Declaration, and the Research Ethics Committee of the Rey Juan Carlos University approved the study with register number 220720153515.A.

### 2.5. Statistical Analysis

As the size of the subsamples was small, the Shapiro-Wilk test was used to verify the homogeneity of the sample. All variables fulfilled the normality criteria except for the Nine-Hole Peg Test; therefore, parametric tests were used for all the other variables. The parametric analysis was performed using the one-factor ANOVA (for more than two groups) and Student's *t*-test. The nonparametric analysis was performed using the Kruskal-Wallis test on independent samples of more than two groups, whereas the Mann-Whitney *U* test was used on two groups. In addition, the effect size of the differences was estimated using Cohen's *d* test transformed into the correlation coefficient (*d*_*r*_) [26], which is interpreted as *d*_*r*_ = 0.20 (low), *d*_*r*_ = 0.40 (medium), *d*_*r*_ = 0.60 (high), and *d*_*r*_ = 0.80 (very high).

Initially, the descriptive data of the sample was obtained and classified by subtypes in order to observe the frequency of the categorical variable and the mean, the standard deviation, and the median of the remaining continuous variables. Secondly, the dependent variables were analyzed according to the previously described comparative groups to examine the manipulative dexterity of all subjects included in the study in a cross-sectional manner as comparative groups and according to the type of MS and the more affected side. Lastly, to analyze possible correlations and predictive variables among the different variables, Pearson's correlation test was used together with simple linear regression.

The data analysis was performed using the SPSS v.25 program, adding a syntax for the estimation of the effect size.

## 3. Results

The initial sample was 35 participants; however, five patients left the study due to a flare-up of the disease, family problems, or lack of collaboration. The final sample comprised 30 patients, 14 men and 16 women, with a mean age of 45 ± 8.11 years. The sample was fairly homogeneous regarding the cognitive level and the state of disability measured using the EDSS.  Table 1 features the descriptive data of the sample by subtypes.

The initial analysis described the manipulative dexterity according to the different types of MS, revealing manipulative dexterity deficits according to normative data. Thus, patients with all kinds of MS obtained lower scores for skill levels compared to the norm. The lowest values were those of the PPMS group. However, the analysis of differences between subtypes (Table 2) did not reveal statistically significant intragroup differences, except in the case of the PPT test, where differences between groups were found regarding manipulative dexterity. Thus, we observed differences regarding the effect size of practically all the variables analyzed for both manipulative dexterity (PPT, NHPT, and BBT) and the self-perception of ADLs (ABILHAND), for which high values (*d*_*r*_ = 0.72) and very high values (*d*_*r*_ = 1.46) were obtained.

When analyzing the data by groups according to the more affected side (Table 3) (whether this be dominant or nondominant), once again, significant intragroup differences were found. This result was particularly evident in the PPT test for the right UL (*p* < 0.00). No other significant difference was found in the remaining tests for manipulative dexterity, or in the test for the self-perception of ADL. Nonetheless, as occurring in the previous comparative groups, when analyzing the effect size, in several variables, a high value (*d*_*r*_ = 0.60) and very high value were observed (*d*_*r*_ = 1.32) regarding the clinical implication of the same. The Pearson correlation coefficient was used on the tests for manipulative dexterity, revealing high correlations (*p* < 0.001) among all these tests (Table 4). In contrast, the ABILHAND test only revealed a significant correlation with the PPT test, overall. Regarding the correlation between the years of evolution, as well as the age, gender, and manipulative dexterity, no significant correlations were found. In order to further our knowledge on the possible influence of manipulative dexterity scores on the dimensions of ABILHAND, multiple linear regression models were performed, adjusted for the variables sex, years of evolution, and type of MS.  Table 5 displays the results of the multiple linear regression model, which reveals that none of the variables considered showed a statistically significant effect for dexterity, except the PPT and years of evolution, which showed a statistically significant and positive effect. Thus, the PPT predicts up to 21.8% of the variance of the ABILHAND, which measures self-efficacy of dexterity in ADLs.

## 4. Discussion

The purpose of the present study was to describe the differences among subtypes of MS regarding manipulative dexterity, as well as to detect the possible correlations and predictors between manual dexterity and perceived dexterity during ADLs.

According to our findings, a manipulative dexterity deficit was observed in participants with all subtypes of MS, although this deficit was greater in individuals with PPMS, which is in line with a previous report [8]. Furthermore, this decline does not depend on the subtype of MS, as we did not observe statistically significant differences by subgroup, except in the PPT dominant hand test. A possible reason for this may be that although each subgroup has a different disease evolution, all subtypes experience the same symptoms at some point, including manipulative dexterity deficit, without statistically significant differences. This correlates with findings by other authors, who did not specify differences between subtypes [18]. Nonetheless, even though no significant differences are found, our results point to an impact factor among the different groups, where the main differences are found in the PPMS and SPMS subtypes. Studies along these lines indicate that different stages for the disease exist, meaning that quality of life varies substantially [16, 17, 27].

Additionally, regarding manipulative dexterity, statistically significant differences between groups were not found according to the most affected side, except the PPT dominant hand test. Although Lamers et al. [5] indicated that the nondominant UL tends to obtain poorer scores, our results are in agreement with other studies, such as Bertoni et al. or the study by Lamers et al. [9] in 2015, which confirmed a bilateral impairment in people with MS. Besides, Cattaneo et al. [28] did not specify the affected side for limited hand dexterity; however, they reported participation restrictions in home activities, implying a bilateral use of both extremities. This finding is clinically significant, because a bilateral impairment leads to a significant decrease in home and productive activities, which will gradually decrease quality of life.

Regarding our last aim, to analyze the correlations between dexterity variables and self-perception and to identify possible predictors of functionality, different studies [14, 28, 29] have highlighted the existence of a significant relationship between manipulative dexterity and participation in ADLs. Considering the importance of this relationship and the relevance of participation in ADLs, our study researched whether the same situation happens with the perception that people with MS have regarding their manipulative dexterity in ADLs and their true skills, measured using objective scales. Our findings show that there is no correlation between what people perceive and their manipulative dexterity measured with objective outcomes. This finding is relevant for clinical practice, because if people with MS perceive themselves as being worse than they actually are, this will lead to a decrease in participation in outdoor and indoor activities, even social activities. Other studies [30–32] state that the lack of participation in these activities leads to a reduced self-esteem, life satisfaction, and mental health status. Therefore, the lower number of activities may further affect the level of physical capacity, leading to a further reduction in participation [33].

The manual dexterity of people with MS has long concerned researchers who, for many years, have attempted to determine the predictive factors of the disease progression, as well as the most ideal means for improving patients' quality of life. In 2002, O'Hara et al. [34] evaluated the effectiveness of professionally guided self-care for people with MS living in the community, emphasizing the importance of ADLs and the impact that manual dexterity has for the performance of the same. More recent studies by Feys et al. [35] in 2004, Gharagozli et al. [36], or Guclu-Gunduz et al. [37] in 2012 follow a similar line. The results of our work regarding the impact of the disease on manipulative dexterity concur with the findings of these previous reports. In 2012, Kamm et al. [38] associated manual dexterity difficulties with apraxia in patients with MS. This factor equaled a worse prognosis for the progression of the disease and, consequently, for the resulting disability. Other authors have related manual dexterity issues with visual, sensory, or motor disorders although, to date, there is no consensus regarding what factors would justify their presence. In 2015, Ghandi Dezfuli et al. [39] evaluated the manual dexterity of 60 patients using the PPT test and discovered that for 60.5% of the sample, the manual dexterity of the dominant hand and the duration of the disease are both predictors of the disability status; therefore, a reduced dexterity of the dominant hand is a factor of disability. Our results, regarding predictors of functionality, provide an important contribution to understanding the self-perceived manipulative dexterity. However, none of the dexterity outcome measure variables displayed a relationship with self-perception, except the PPT variable, predicting only 21.8% self-efficacy for dexterity in ADLs. This means that although a person with MS may have obtained good scores for manipulative dexterity, what they perceive may be different. Thus, emotional components, such as anxious or depressive symptoms, are extremely important factors, as depression will probably affect a person's physical activity and may lead to worse participation and social integration, as stated by Battalio et al. [40]. This is along the lines of a previous study by Schmitt et al. [41], affirming that self-perceived efficacy plays a very important role in the individual adjustment towards MS in several areas of functional outcome, beyond what can be explained via disease variables alone. It is highly important to address clinical interventions with people with MS not only directed at motor or cognitive aspects, or even quality of life, but also towards the person's self-perceived dexterity.

The present study presents important limitations. The sample size limits the possibility of reaching generalizable conclusions. Furthermore, the number of intragroup participants does not represent the same number of participants; therefore, the groups are unbalanced. However, the difficulty of recruiting older people should be taken into consideration. In a 10-year prospective study by Conradsson et al. [42] in 2018, the sample size of the subgroups was reduced to 89 people with RRMS and 12 with PPMS with moderate impairment.

The findings of this study present valuable clinical implications. No significant differences were observed among the different types of MS, although there were differences in the effect size, and in dexterity impairments. The observed deficits are in line with previous reports [28]; however, we feel that it is a key to focus on a person's perception of their skill level besides their motor problems. The evaluation of manual dexterity in people with MS, and the impact on the perceived efficacy in ADLs, must be considered as prognostic factors for progression of the disease.

## 5. Conclusions

In conclusion, our results indicate that manipulative dexterity deficits exist in all types of MS. Also, this study suggests that self-perception should be considered in the rehabilitation of people with MS, as a possible predictor of functionality. Future research with a larger sample size should consider other variables that may be involved in the manipulative dexterity deficits seen in patients with MS. This will support further research on targeted interventions to improve these deficiencies, while at the same time promote an improvement of the quality of therapy provided.

## Figures and Tables

**Table 1:** Descriptive data of the continuous and categorical variables of the sample by MS subtypes (*n* = 30).

	Relapsing-remitting (*n* = 15)		Primary progressive (*n* = 8)		Secondary progressive (*n* = 7)	
Continuous variables	*M* ± SD	Median (q1-q3)	*M* ± SD	Median (q1-q3)	*M* ± SD	Median (q1-q3)
Age	43 ± 8.92	42 (35-53)	48 ± 5.05	50.5 (43.2-53)	48 ± 5.05	53 (41-53)
Years of evolution	8.8 ± 4.84	8 (5-10)	5.4 ± 4.60	4 (1.2-10.5)	16.78 ± 7.77	17 (11-26)
EDSS	4.2 ± 1.99	5 (2.37-6)	6.1 ± 0.62	6 (6-6.5)	5.5 ± 1.02	6 (5.3-6)
MoCA	24.2 ± 3.93	25 (23-28)	25.6 ± 3.46	26 (23.5-28.5)	26.5 ± 3.4	27 (23-30)
Categorical variables	Fr (%)		Fr (%)		Fr (%)	
*Gender*						
Female	9 (60%)		4 (50%)		3 (42.9%)	
Male	6 (40%)		4 (50%)		4 (57.1%)	
*Studies*						
Basic	2 (13.3%)		2 (25%)		0 (0%)	
High school	8 (53.3%)		4 (50%)		2 (28.6%)	
University	15 (33.3%)		2 (25%)		5 (71.4%)	
*Upper limb dominance*						
Right	14 (93.3%)		7 (87.5%)		7 (100%)	
Left	1 (6.7%)		1 (12.5%)		0 (0%)	
*More affected side*						
Right	7 (46.7%)		3 (37.5%)		3 (42.9%)	
Left	8 (53.3%)		5 (62.5%)		4 (57.1%)	

**Table 2:** Comparison among subsamples according to the type of multiple sclerosis.

	Relapsing recurrent (*n* = 15)	Primary progressive (*n* = 8)	Secondary progressive (*n* = 7)		
	Median (q1-q3)			*p* value	*d*_*r*_
PPT_D	9 (7-13)	6 (3.5-9.7)	10 (8-13)	0.02	1.46
PPT_ND	8 (7-11)	8 (1.25-10.75)	10 (7-12)	0.33	0.72
PPT bilateral	6 (4-9)	2 (0.25-8.25)	7 (6-8)	0.10	1
PPT assembly	17 (14-23)	10.5 (9-17.5)	21 (19-26)	0.11	1.01
PPT total	25 (18-34)	18 (10.5-25)	28 (22-33)	0.03	1.2
NHPT_D	23.6 (19.9-31.7)	30.3 (22.6-107.8)	25.2 (24.3-27.8)	0.73	0.73
NHPT_ND	28 (23.8-33.5)	43.5 (26.3-60.8)	30 (21-35)	0.11	0.14
BBT_D	51 (35-59)	44 (21.25-48.7)	46 (43-55)	0.19	0.37
BBT_ND	45 (35-53)	39.5 (17.2-44.7)	50 (38-64)	0.08	0.91
ABILHAND	1.82 (1.3-2.7)	1.5 (0.4-2.4)	3.2 (1.1-4.4)	0.09	0.96

**Table 3:** Comparison among subsamples according to the most affected side, dominant or nondominant.

	More affected side—dominant (*n* = 13)	More affected side —nondominant (*n* = 17)		
	Median (q1-q3)		*p* value	*d*_*r*_
PPT_D	7 (5.5-8.5)	13 (9-13.5)	0.03	1.32
PPT_ND	8 (7-11.5)	9 (5-11.5)	0.27	0.33
PPT bilateral	6 (3-8)	7 (3.5-9)	0.92	0.10
PPT assembly	16 (9-19)	21 (10.5-26)	0.10	0.60
PPT total	19 (17.5-26)	28 (18.5-33.5)	0.43	0.40
NHPT_D	31.7 (24-39)	22.5 (20.4-25.5)	0.81	0.73
NHPT_ND	33.3 (26.6-35.7)	25.4 (22.3-43.9)	0.46	0.18
BBT_D	36 (32-50.5)	49 (44-57)	0.08	0.60
BBT_ND	40 (35-50.5)	45 (39.5-54.5)	0.94	0.00
ABILHAND	1.8 (0.96-2.85)	1.9 (1.2-3.4)	0.46	0.42

**Table 4:** Correlations among the different variables of manipulative dexterity and perception of dexterity in activities of daily living.

	PPT_D	PPT_ND	PPT bilat	PPT	PPT assembly	NHPT_D	NHPT_ND	BBT_D	BBT_ND	ABILHAND
PPT_D	1	0.334	0.567^∗∗^	0.796^∗∗^	0.737^∗∗^	-0.655^∗∗^	-0.488^∗∗^	0.729^∗∗^	0.470^∗∗^	0.294
PPT_ND	0.334	1	0.542^∗∗^	0.776^∗∗^	0.609^∗∗^	-0.374^∗^	-0.540^∗∗^	0.304	0.661^∗∗^	0.262
PPT bilat	0.567^∗∗^	0.542^∗∗^	1	0.853^∗∗^	0.732^∗∗^	-0.429^∗^	-0.477^∗∗^	0.433^∗^	0.641^∗∗^	0.330
PPT total	0.796^∗∗^	0.776^∗∗^	0.853^∗∗^	1	0.858^∗∗^	-0.609^∗∗^	-0.623^∗∗^	0.613^∗∗^	0.728^∗∗^	0.364^∗^
PPT assembly	0.737^∗∗^	0.609^∗∗^	0.732^∗∗^	0.858^∗∗^	1	-0.576^∗∗^	-0.538^∗∗^	0.682^∗∗^	0.720^∗∗^	0.311
NHPT_D	-0.655^∗∗^	-0.374^∗^	-0.429^∗^	-0.609^∗∗^	-0.576^∗∗^	1	0.857^∗∗^	-0.622^∗∗^	-0.79^∗∗^	-0.275
NHPT_ND	-0.488^∗∗^	-0.540^∗∗^	-0.477^∗∗^	-0.623^∗∗^	-0.538^∗∗^	0.857^∗∗^	1	-0.413^∗^	-0.580^∗∗^	-0.157
BBT_D	0.729^∗∗^	0.304	0.433^∗^	0.613^∗∗^	0.682^∗∗^	-0.622^∗∗^	-0.413^∗^	1	0.648^∗∗^	0.271
BBT_ND	0.470^∗∗^	0.661^∗∗^	0.641^∗∗^	0.728^∗∗^	0.720^∗∗^	-0.479^∗∗^	-0.580^∗∗^	0.648^∗∗^	1	0.277
ABILHAND	0.294	0.262	0.330	0.364^∗^	0.311	-0.275	-0.157	0.271	0.277	1

Note: ^∗^*p* < 0.005; ^∗∗^*p* < 0.001.

**Table 5:** Effect of manipulative dexterity on self-perceived dexterity.

	ABILHAND		
	Estimate (SE)	*t*	Pr(>∣*t*∣)
(Intercept)	-0.461 (0.957)	-0.481	0.6341
PPT total	0.079 (0.034)	2.314	0.05
Years of evolution	0.077 (0.037)	2.047	0.028
*R* ^2^ (%)	21.8		
Model	ABILHAND = −0.461 + 0.077∗years of evolution + 0.079∗PPT total		

PPT = Purdue Peg Test; SE = standard error.

## Data Availability

The availability of data from this study has been included in a confidential database only used for this study.

## Abbreviations

M: Mean
SD: Standard deviation
q1-q3: Percentiles
Fr: Frequency
MoCA: Montreal Cognitive Assessment, screening test
PPT_D: Purdue Pegboard Test, manipulative dexterity test for the dominant upper limb
PPT_ND: Purdue Pegboard Test, manipulative dexterity test for the nondominant upper limb
PPT bilateral: Purdue Peg Test, manipulative dexterity test for both upper limbs
PPT total: Purdue Peg Test, manipulative dexterity test, total score
NHPT_D: Nine-Hole Peg Test, manipulative dexterity test in the dominant upper limb
NHPT_ND: Nine-Hole Peg Test, manipulative dexterity test on the nondominant upper limb
BBT_D: Box and Block Test, manipulative dexterity test on the dominant upper limb
BBT_ND: Box and Block Test, manipulative dexterity test on the nondominant upper limb.
